# Comparison between two self-guided tinnitus pitch matching methods

**DOI:** 10.3389/fnagi.2023.1095178

**Published:** 2023-01-25

**Authors:** Jose L. Santacruz, Emile de Kleine, Pim van Dijk

**Affiliations:** ^1^Department of Otorhinolaryngology/Head and Neck Surgery, University Medical Center Groningen, University of Groningen, Groningen, Netherlands; ^2^Graduate School of Medical Sciences (Research School of Behavioral and Cognitive Neurosciences), University of Groningen, Groningen, Netherlands

**Keywords:** tinnitus, pitch, matching, self-guided, hearing loss

## Abstract

**Introduction:**

Tinnitus pitch matching is a procedure by which the frequency of an external sound is manipulated in such a way that its pitch matches the one of the tinnitus. The correct measure of the tinnitus pitch plays an important role in the effectiveness of any sound-based therapies. To date, this assessment is difficult due to the subjective nature of tinnitus. Some of the existing pitch matching methods present a challenge for both patients and clinicians, and require multiple adjustments of frequency and loudness, which becomes increasingly difficult in case of coexisting hearing loss. In this paper, we present the comparison in terms of reliability between two self-guided pitch matching methods: the method of adjustment (MOA) and the multiple-choice method (MCM).

**Methods:**

20 participants with chronic tinnitus and hearing loss underwent the two assessments in two different sessions, 1 week apart. Measures of intraclass correlation (ICC) and difference in octaves (OD) within-method and within-session were obtained.

**Results:**

Both methods presented good reliability, and the obtained values of ICC and OD suggested that both methods might measure a different aspect of tinnitus.

**Discussion:**

Our results suggest that a multiple-choice method (MCM) for tinnitus pitch matching is as reliable in a clinical population as more conventional methods.

## 1. Introduction

Tinnitus is often defined as the perception of sound without an external source. Several studies have reported the tinnitus prevalence in the population, which ranges from 5.1% to 42.7% (McCormack et al., 2016). One of the main challenges of health care when addressing tinnitus is the large heterogeneity of its symptoms and etiologies (Langguth, 2011), making it improbable that a specific therapy would be suitable for every patient (Hall et al., 2019). Some authors have highlighted the importance of personalized treatments, which are prescribed according to the physiological mechanisms that underlie each individual’s symptoms. The most frequent comorbidity of tinnitus is hearing loss which, in the case of the Dutch population, has an association with an odds ratio of 8.5 (Schubert et al., 2021).

There is an increasing interest in sound-based therapies for tinnitus treatment (e.g., Henry et al., 2008; Hobson et al., 2012; McNeill et al., 2012; Shekhawat et al., 2013; Searchfield et al., 2017). The most common sound-based tinnitus therapy by far are hearing aids, and it has been estimated that up to 90% of the tinnitus population may benefit from their use (Henry et al., 2015). Hearing aids increase the volume of external sounds, improving the communication of users. They may help to reduce other tinnitus symptoms like stress or anxiety, but also mask or provide distraction from tinnitus (Sereda et al., 2015). Nevertheless, patients differ with respect to many audiological characteristics, such as the degree of hearing loss, the tinnitus pitch and loudness, the factors that influence their tinnitus or their psychological response to the tinnitus percept (Schaette et al., 2010; Cederroth et al., 2019).

The potential dependency of the tinnitus pitch and the effectiveness of a sound-based therapy has motivated the development of different pitch-based treatments. Examples of these are the vagus nerve stimulation combined with a sound stimulus (De Ridder et al., 2015), tailor-made notch noise training (Stracke et al., 2010), notch filter amplification (Marcrum et al., 2021), harmonic sound therapy (Mahboubi et al., 2012), phase-shift sound therapy (Heijneman et al., 2012), or different discrimination/attention tasks focused on re-adjusting the attention to the tinnitus percept (Hoare et al., 2010; Wise et al., 2015).

Sound-based therapies are often fine-tuned to the pitch of the tinnitus (Hoare et al., 2014). A procedure well-known in the tinnitus field, is tinnitus matching, where the frequency of an external sound is manipulated such that its pitch matches that of the tinnitus (Henry and Meikle, 2000). Although pitch matching is part of the standard audiological assessment of a tinnitus clinic, its reliability is often questioned due to its self-reported nature and the large variability between consecutive sessions (Hoare et al., 2014), which can even vary over 2 octaves (Henry et al., 2004). It remains unclear whether these variations are the result of the patients’ difficulties when performing the tests or whether they reflect a change of the percept between sessions (Penner and Bilger, 1992). Even though clinicians have to rely on patients’ feedback when performing a pitch matching test, the procedure does not entirely resemble a “black box.” Many authors have investigated the relationship between the audiogram and the tinnitus pitch and, more specifically, several instances can be found in the literature where authors theorize on the link between audiogram edge and pitch (Schaette and Kempter, 2009; Moore, 2010; Jain et al., 2021). However, there seems to be a broader consensus on the relationship between the pitch and the whole frequency region of hearing loss (Norena et al., 2002; Roberts et al., 2006; Schecklmann et al., 2012; Jain et al., 2021).

The literature reports plenty of different approaches to carry out the pitch matching, and their performance have been extensively compared with each other (Tyler and Conrad-Armes, 1983; Penner, 1995; Henry et al., 2004; Neff et al., 2019). Some of these methods consist of several steps of choices where the distance in frequency between the presented tones is narrowed step by step, just as in the case of the two-alternative forced-choice method (2AFC; Penner and Bilger, 1992) or the forced-choice double staircase (FCDS; Henry et al., 2013). Other methods, such as the likeness rating (LR; Norena et al., 2002), aim to broaden the tinnitus characterization from a single frequency to a wider spectrum by means of comparisons between the subject’s percept and several pure tones of different frequencies. Unlike these approaches, which are usually based on the interaction between audiologist and patient through a series of questions and adjustments, the method of adjustment (MOA) allowed subjects to self-guide the test by using a computer interface or a noise generator and dial (Tyler and Conrad-Armes, 1983; Henry et al., 2004). The MOA involves the constant presentation of a stimulus (typically a pure tone or a narrow-band noise) whose frequency and loudness can be controlled by the subject. The finer adaptability of this method might provide a more accurate representation of the subject’s tinnitus. However, the MOA can be difficult to perform for some patients due to a steep slope of their audiogram, which leads to numerous adjustments of the loudness dial (Penner and Bilger, 1992). It is worth mentioning that most pitch matching methods require extra time for the adjustment of the stimulus loudness, despite the fact that pitch-based therapies (as their name suggests) do not usually need loudness data to be implemented.

Due to the above-mentioned reasons, we decided to develop a different pitch matching method and to compare its performance to the MOA. In this paper, we report the reliability of a self-guided multiple-choice method (MCM) for tinnitus pitch matching, and we compare the results to the MOA between sessions. With the MCM, we aim for an easy-to-conduct method, with higher reliability and a user-friendly interface to simplify the procedure.

## 2. Methods

### 2.1. Participants

A total of 20 adult patients of the Otorhinolaryngology Department of the University Medical Center Groningen (UMCG) were recruited to participate in this study between September of 2020 and April of 2021. All of the 20 participants had chronic tinnitus (suffering tinnitus for at least 3 months; Vesterager, 1997) and presented a symmetric hearing loss (≤15 dB difference between both ears at 2, 4 and 8 kHz) with an averaged pure-tone audiometry (PTA at the same frequencies) of at least 30 dB. Excluding tinnitus and hearing loss, participants had no history of either neurological or psychiatric disorders. All participants gave written informed consent to join the study, which was approved by the ethics committee of the University Medical Center Groningen (METc 2018/445).

### 2.2. Questionnaires

After giving written informed consent and prior to being invited to the clinic, participants received by mail a series of questionnaires that were sent back to us with a return envelope. These questionnaires were the Tinnitus Functional Index (TFI; Meikle et al., 2012), the Hyperacusis Questionnaire (HQ; Khalfa et al., 2002) and the European School for Interdisciplinary Tinnitus Research Screening Questionnaire (ESIT-SQ; Genitsaridi et al., 2019). The latter was used to gather demographic data and additional tinnitus characteristics.

### 2.3. Method of adjustment

**Figure 1:**
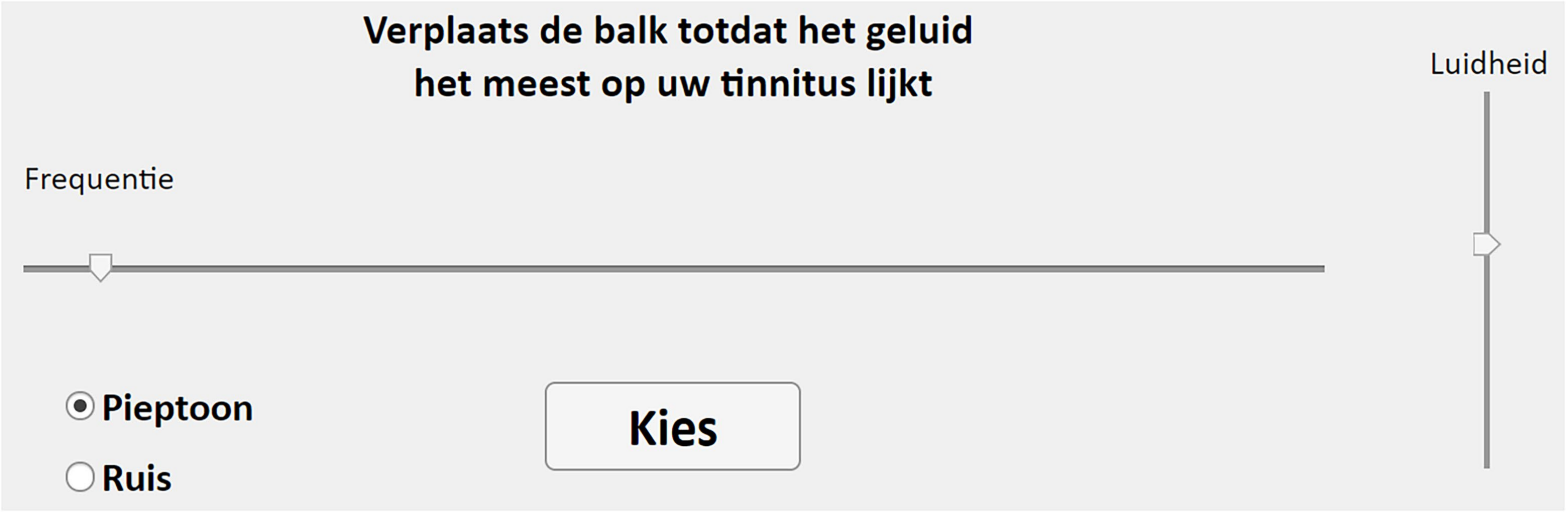
MOA’s interface.

When the process starts, the question *“Hoe klinkt uw tinnitus?”* (“what does your tinnitus sound like?”) appears on the screen, followed by two clickable answers: *“Pieptoon”* (“Beep”) for pure tone and “Ruis” (“Noise”) for narrow-band noise with a bandwidth of ⅓ of an octave. After choosing one of the two, the stimulus is presented initially at 1 kHz and 60 dB SPL, while the interface shows the sentence *“Verplaats de balk totdat het geluid het meest op uw tinnitus lijkt,”* meaning “Move the bar until the sound most resembles your tinnitus” (Figure 1). The subject then can adjust the central frequency and the loudness of the stimulus by using two sliders. The stimulus, which is continuously presented during the entire test, can also be changed between pure tone and noise at this stage. The subject can finalize this stage by pressing the button *“Kies”* (“Choose”), by means of which the frequency of the stimulus is stored. Next, an octave confusion test is performed. For this, the selected frequency is tested against two other stimuli that are centered at an octave below and an octave above, with the three of them presented at the same intensity level. Here, the participant has to choose one out of the three options, which is stored as the final frequency and loudness of the pitch matching process. Onset and offset times of the stimuli were 100 ms. The frequency slider (range from 0.05 to 16 kHz) allows minimum changes in linear steps of 18.5 Hz, and the loudness slider (range from 10 to 95 dB SPL) can be adjusted in steps of 0.81 dB.

### 2.4. Multiple-choice method

**Figure 2:**
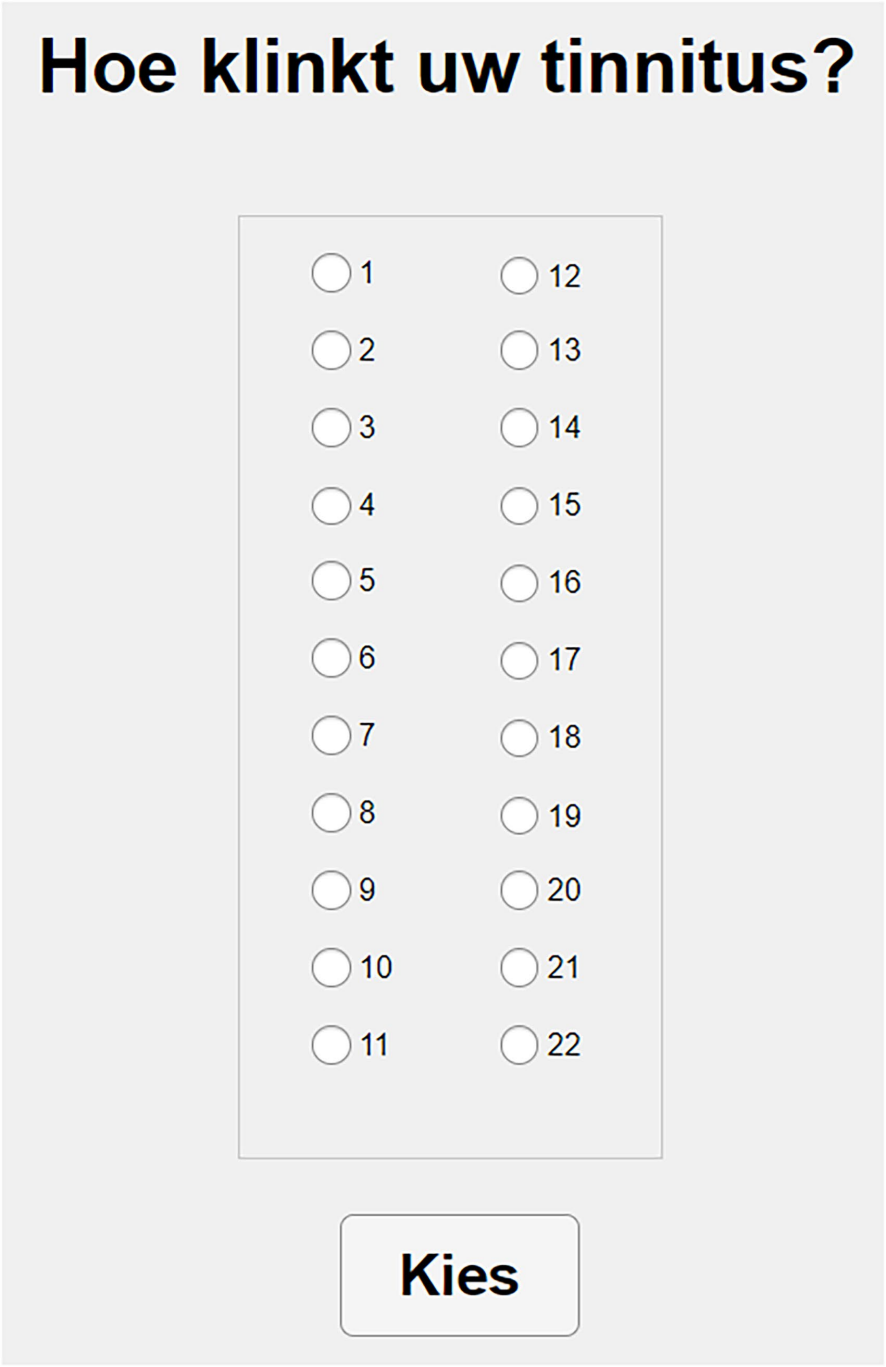
MCM’s interface.

Like MOA, this method starts by asking the participant to choose between noise and pure tone. After the subject chooses one of the two options, the interface shows 22 different clickable buttons that can be activated one by one (see Figure 2). Each of them then presents a stimulus of 1 s duration and centered at the following frequencies: 0.1, 0.3, 0.5, 0.6, 0.7, 0.8, 1, 1.1, 1.2, 1.35, 1.5, 2, 2.5, 3, 3.5, 4, 5, 6, 7, 9, 10, and 12 kHz. Stimuli are presented at a comfortable level and adjusted to the participant’s audiogram, according to the following procedure: the level of each stimulus is adjusted by adding 60 dB of baseline presentation level to the dBs of the nearest frequency available of the audiogram, with a maximum level of 95 dB SPL. Bandwidth of the noise, onset and offset times are identical to MOA.

### 2.5. Procedure

**Figure 3:**
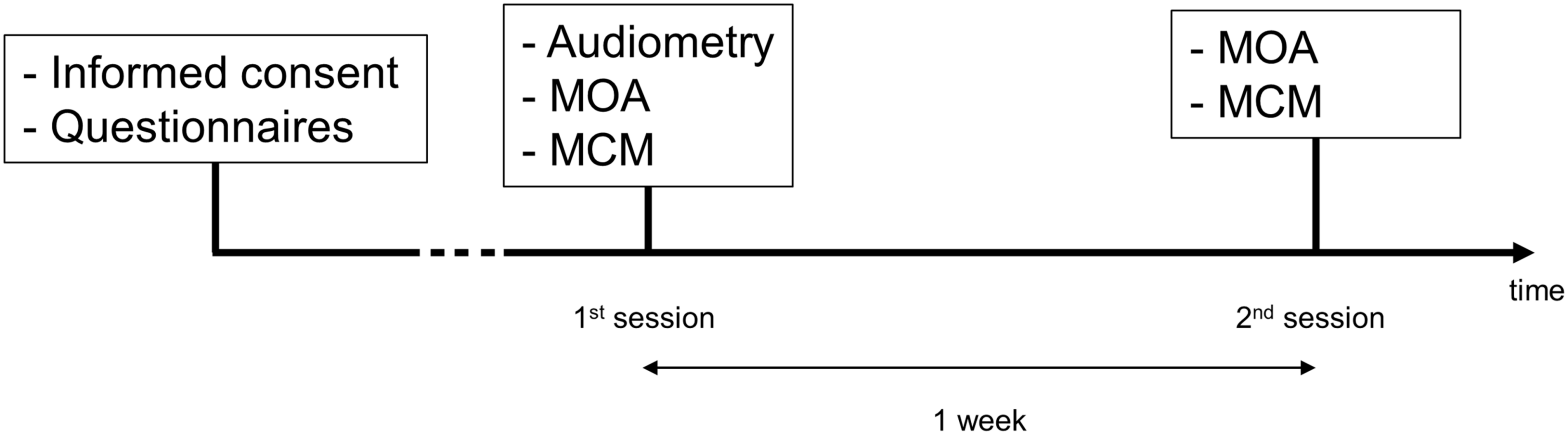
Timeline of the experiment. The measurements are shown in chronological order for each session.

Figure 3 shows the timeline of the experiment. The participants were invited to come to the clinic for two sessions, 1 week apart. Hearing thresholds were measured during the first session with a conventional audiometry at frequencies between 0.125 and 8 kHz in octave steps, as well as 3 and 6 kHz. For this, an audiometer AC40 (Interacoustics) and a pair of TDH39 headphones (Telephonics) were used. All measurements were carried out in sound proof rooms. For the pitch matching procedures, a MOTU UltraLite audio interface and a pair of Sennheiser HD660S headphones wer used. All sounds were delivered monoaurally. In case of unilateral tinnitus, sounds were presented in the contralateral ear. For the bilateral cases, sounds were presented in the best hearing ear.

### 2.6. Analysis

Data was analyzed in R version 4.0.2. Sample size was determined based on a power analysis with an expected reliability of 0.9, a minimum acceptable reliability of 0.65 and a significance level of 0.05 (*α*). Reliability of the two matching methods was estimated by means of several coefficients and measures. Intraclass correlation (ICC) was used to quantify the reliability of each method within and between sessions. ICC was estimated using the package “irr” of R (version 0.84.1). Data were tested for normality by the Shapiro–Wilk Test. Mean frequencies and standard deviations over all participants for both methods between and within sessions were calculated. Moreover, the within-method and within-session differences in octaves were also estimated. Mean loudness and standard deviation of MOA was obtained.

## 3. Results

Demographic characteristics of the participants are presented in Table 1. Hearing thresholds were assessed by estimating a Pure Tone Average (PTA of 2, 4 and 8 kHz) and did not differ significantly between ears. Averaged values of the TFI and HQ scores are shown.

**Table 1:** Demographic characteristics of the participants.

Demographic data	
Number of subjects (*n*)	20
Age (years)	62.2 ± 8.5
*Sex* — *n* (%)	
Male	15 (75)
Female	5 (25)
Average hearing threshold in both ears (dB HL)	51.3 ± 11.6
*PTA (2, 4 and 8 kHz)*	
Left ear	52.0 ± 11.5
Right ear	50.7 ± 12.6
TFI score (0–100)	51.4 ± 16.4
HQ score (0–42)	21.5 ± 7.7

Mean values and standard deviation are presented, unless stated otherwise.

**Figure 4:**
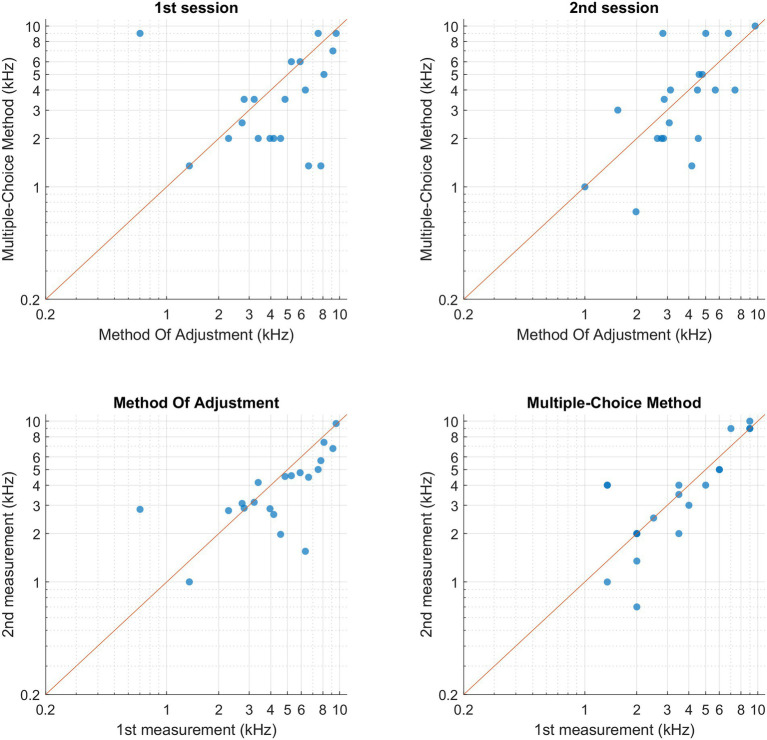
Participant’s pitch-matching results for both methods and both sessions, each data point represents one participant. (Upper-left corner) Comparison between bothmethods within the 1st session. (Upper-right corner) Comparison between both methods within the second session. (Bottom-left corner) Comparison within MOA between both sessions.(Upper-right corner) Comparison within MCM between both sessions.

**Figure 5:**
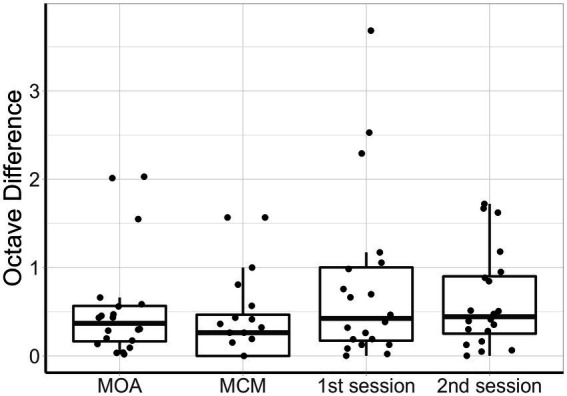
Box plots of the difference in octaves, from left to right: MOA within-method, MCM within-method, 1st session between methods, 2nd session between methods. For each boxplot, the data points represent individual participants.

Figure 4 shows the individual pitch-matching results during both sessions and using both MOA and MCM. Normality of the data could not be assumed for MCM. The reliability measures and pitch matching averages of both methods are represented in Table 2. When comparing the two methods, the intraclass correlation coefficient was higher for MCM compared to MOA. However, the overlap between the two confidence limits of both ICCs indicated that there was no significant difference between both methods. There was no significant difference in the averaged tinnitus pitch between both methods. The mean octave difference (OD) between the two sessions was calculated for both methods, no significant difference was found. The within-method and within-session individual’s ODs are shown in Figure 5.

**Table 2:** Averaged pitch matching results and reliability measures.

Comparison	ICC (95% CI)	Freq (kHz)	OD	Loudness (dB SPL)
*Between sessions, within-method*				
Method of Adjustment (MOA)	0.77 (0.49–0.90)	4.4 ± 2.4	0.53 ± 0.60	78 ± 12
Multiple-Choice Method (MCM)	0.92 (0.81–0.97)	4.0 ± 2.8	0.39 ± 0.48	–
*Within-session, between methods*				
Session 1	0.43 (0.02–0.73)	4.4 ± 2.7	0.80 ± 0.97	
Session 2	0.62 (0.25–0.83)	4.1 ± 2.5	0.62 ± 0.55	

ICC, intraclass correlation; OD, difference in octaves. Mean values and standard deviations are presented, unless stated otherwise.

## 4. Discussion

In this study, we compared two self-guided methods to measure the tinnitus pitch in 20 participants with chronic tinnitus. The participants used the both methods MOA and MCM to measure their tinnitus pitch in two sessions, 1 week apart. The comparison was made by means of reliability, mean frequencies and octave difference between and within sessions.

Both methods presented very good reliability. However, due to the relatively large confidence intervals of the ICC, it is not possible to determine which one of the methods is more reliable. Nonetheless, MCM presented an ICC ≥ 0.9, which is considered the required standard of a tool used for clinical decision making for individual patient data (Kottner et al., 2011). MOA presented an ICC ≥ 0.7, indicating good agreement between measures for group data (Nunnally and Bernstein, 1994). In terms of octave difference, no significant difference was found between both methods as a result of the spread of the data.

A previous study compared three different pitch matching methods including the MOA, for which they obtained analogous results of reliability (Neff et al., 2019). In this study, the authors mentioned the potential bias of the participants in the decision-making due to the initial presentation of the stimulus of this method (1 kHz in our case), which explains differences in pitch between methods. However, the authors did not report the standard deviation of the frequency selection. In our case, despite no significant difference in frequency was found between methods, we also suspect that the starting frequency can play a role in the procedure. This potential bias is avoided in the MCM, which is not initialized with any stimulus. However, some subjects have the tendency of starting the matching from the first option, which corresponds to the lowest frequency available. Future implementations could prevent this by removing the numbers from the buttons and keeping the same sequence of frequencies.

Pitch-dependent sound-based therapies such as the tailor-made notch noise training (Stracke et al., 2010), the notch filter amplification (Marcrum et al., 2021) or the harmonic sound therapy (Mahboubi et al., 2012) are based on narrow-band approaches which commonly use a bandwidth of half or a third of an octave. Consequently, frequency resolution might not be the most important characteristic of a pitch matching procedure. Instead, a self-guided method that allows the subject to choose the closest available option without having to constantly adapt the volume of the stimulus (as in the case of MOA), might be a practical solution for a clinical environment. Another advantage that the MCM presents is the automatic adaptation of the loudness of the stimuli to the hearing profile of the subject. In the case of MOA, patients with high-frequency tinnitus often have trouble adjusting the loudness of the stimulus due to the abrupt decrease of their audiogram, which could be solved by using loudness correction. The MCM addresses this issue so the subject can focus only on the frequency of the sound. Future implementations of the method could adjust the intensity in a more cautious way for high frequencies, following a half-gain rule as in hearing aids fitting (Lybarger, 1963). Moreover, the MCM can be implemented on mobile devices such as smartphones or tablets, which have the potential to be used for hearing diagnosis after the corresponding validation (Wunderlich et al., 2015; Hauptmann et al., 2016; De Wet Swanepoel et al., 2019).

Unfortunately, the test duration was not recorded. This limitation prevents us from claiming that one of the methods has significantly lower duration than the other one. Nevertheless, by observing the participants during the experiment, we noted shorter durations during pitch matching with the MCM than with the MOA. Additionally, it’s worth mentioning the fact that the order of test procedure was not randomized, which could potentially result in a learning effect when performing the second test. Another limitation that both methods had during the experiment is the constraint of 95 dB HL as the maximum level of presentation of the stimulus as a safety measure. A subject whose tinnitus’ loudness is above that level is likely to choose the closest audible frequency during the matching procedure. For presentations within the extended high frequency range in the MCM, a similar problem can be seen: since the stimuli were adjusted to the audiogram, and this was measured up to 8 kHz, presentations for extended high frequencies will not be perceived equally loud by participants with high frequency hearing loss. An extended high frequency audiometry could mitigate this issue. Previous comparisons between pitch matching methods used repeated measurements in one single session, which might not be a sufficient time interval to reveal changes in cases of fluctuating tinnitus (Neff et al., 2019). Instead of several measurements in one session, we opted for measuring in 2 different sessions, 1 week apart. The fact that the obtained within-methods ICC values and OD values are higher and lower, respectively, than the between-method ICC and OD, suggests that each method is consistently measuring a different aspect of tinnitus. However, this aspect or feature differs between both methods, hence the higher between-methods OD and lower between-methods ICC. Based on these results, we cannot conclude whether the differences between the two sessions are a result of changes in the tinnitus or an overall difficulty that subjects may have to match an external stimulus to their tinnitus. In addition to this, it is noteworthy the difference in step sizes between both methods, which can affect the reliability results.

To conclude, our results suggest that a multiple-choice method (MCM) for tinnitus pitch matching is as reliable in a clinical population as more conventional methods such as the method of adjustment (MOA). This self-guided approach can be easily implemented on mobile devices. Due to the limited number of response options and the only requirement of having to include the subject’s hearing threshold in advance, the MCM has the potential to speed-up the matching process.

## Data availability statement

The raw data supporting the conclusions of this article will be made available by the authors, without undue reservation.

## Ethics statement

The studies involving human participants were reviewed and approved by METc UMC Groningen. The patients/participants provided their written informed consent to participate in this study.

## Author contributions

JS, EK, and PD conceived and planned the experiments, contributed to the interpretation of the results. JS carried out the experiments, analyzed the data, and took the lead in writing the manuscript with input from all authors. All authors contributed to the article and approved the submitted version.

## Funding

This project has received funding from the European Research Council (ERC) under the European Union’s Horizon 2020 research and innovation programme (grant agreement no. 722064, ESIT) and the Heinsius Houbolt Foundation.

## Conflict of interest

The authors declare that the research was conducted in the absence of any commercial or financial relationships that could be construed as a potential conflict of interest.

## Publisher’s note

All claims expressed in this article are solely those of the authors and do not necessarily represent those of their affiliated organizations, or those of the publisher, the editors and the reviewers. Any product that may be evaluated in this article, or claim that may be made by its manufacturer, is not guaranteed or endorsed by the publisher.
